# Techno-functional properties and structural changes of pork myofibrillar protein with faba bean protein isolate at various pH levels

**DOI:** 10.5713/ab.260446

**Published:** 2026-09-01

**Authors:** Geon Ho Kim, Koo Bok Chin

**Affiliations:** 1 Department of Animal Science, Chonnam National University, Gwangju, Korea

**Keywords:** Faba Bean Protein Isolate, Gelation, pH Condition, Pork Myofibrillar Protein, Structural Properties

## Abstract

**Objective:**

This study was performed to evaluate the effect of faba bean protein isolate (FBPI) on the techno-functional properties and the structural changes of the pork myofibrillar protein gels (MPGs) at different pH levels.

**Methods:**

Control (without FBPI) and FBPI-added treatments with pH adjusted to 6.00, 6.25, or 6.50 were prepared: control groups (C6.00, C6.25, and C6.50) and FBPI-treated groups (F6.00, F6.25, and F6.50), respectively. In order to evaluate the techno-functional properties and structural changes in the protein matrix, cooking yield, gel strength, protein surface hydrophobicity, sulfhydryl group, electrophoretic protein profile, and microstructure were assessed.

**Results:**

Cooking yield and protein surface hydrophobicity increased with pH; values were higher in C6.50 (91.3% and 31.3 μg) than in C6.00 (86.9% and 26.9 μg). Shear stress values remained consistent among FBPI-treated groups, whereas those of the control groups tended to increase with the adjusted pH level. Electrophoretic protein profiles revealed two distinct bands at 42 and 50 kDa in FBPI-added treatments, which were absent in the control groups; the intensity of these bands increased with the pH level. The F6.50 group presented a more compact three-dimensional protein network, and the increased intensities of the 42–50 kDa bands, corresponding to 7S and 11S globulins, correlated with the structural compaction of the gel matrix.

**Conclusion:**

FBPI improved the techno-functional properties of MPGs, regardless of the adjusted pH levels. In addition, the incorporation of FBPI into MPG was associated with pH-dependent structural changes in the protein and gel network, as observed by the protein profile and microstructure.

## INTRODUCTION

Myofibrillar proteins (MPs) are salt-soluble proteins that constitute skeletal muscles [1]. They are key functional factors that contribute to the water-holding capacity (WHC), gelation, and emulsification of meat products, and these techno-functional properties are directly influenced by the state in which the MPs interact with water [2]. The strong structure–functionality relationship of MPs determines the quality characteristics of muscle foods [3]. For this reason, purified MPs have been widely used as model protein systems for developing desirable meat products.

The pH affects the solubilization of MP, and MP solubility directly affects other techno-functional properties. Furthermore, MP solubilization is a mechanism that influences the development of the quality of processed meats by forming interactions between protein and water via hydrogen bonds and is markedly affected by pH [4]. Myosin has an important role in the structure of meat products because it is the predominant protein responsible for the overall emulsification performance of MP. The myosin-dependent emulsification theory suggests that myosin heads bind rapidly at the oil-water interface to form a thick interfacial film. However, the myosin head region may unfold into a disordered state due to changes in the pH, resulting in the loss of its ability to form a network [5]. Because MP is sensitive to changes in pH that can alter its pI, the incorporation of functional ingredients can be considered to achieve the desired characteristics of the final product as gelling or emulsifying agents [6]. Several studies demonstrated the effects of stabilizers on the gelling and emulsifying properties of MP under various pH conditions. Niu et al. [2] reported the potential of combined acid-treated soy protein isolate (SPI) with improvements in the techno-functional properties as an ingredient of meat products. Hong et al. [5] evaluated microbial transglutaminase (MTGase) and calcium alginate as gelling agents for pork MPs and showed that these could improve gel formation and emulsion stability under different pH conditions. Pan et al. [6] evaluated the textural properties and WHC of pork MP added with flaxseed gum at various pH values. These studies revealed that the properties of the muscle system are affected by pH-dependent techno-functional and rheological properties of food binders and hydrocolloids as gelling and emulsifying agents.

Faba bean (*Vicia faba* L.) is a sustainable high-protein food source, containing abundant lysine and globulin-type storage proteins, which has promoted its utilization as a functional ingredient in various food applications [7]. Furthermore, faba bean protein isolate (FBPI) has techno-functional properties such as solubility, emulsifying activity, and oil-holding capacity comparable with those of commercial isolates from soy, pea, and whey [8].

The pH condition is an important factor when FBPI is to be used for muscle food application. The rheological properties of faba bean may also be sensitive to the pH, similar to those of MP. Langton et al. [9] observed differences in the rheological properties and microstructures of gels made with faba bean protein extract at pH 5.00 and 7.00. They reported that the difference in gel structure might be explained by pH-dependent changes in the solubility of the 7S and 11S fractions, with higher pH increasing solubility and altering the onset temperature heat-induced network formation. Accordingly, gels formed at lower pH (5.00) exhibited a coarser structure, whereas those formed at higher pH (7.00) showed a finer network. Similarly, Kimura et al. [10] reported that the physicochemical properties and structures of the 7S and 11S fractions from faba bean were influenced by hydrophobic interactions due to different pH levels.

In order to identify the potential of faba bean as an extender and binder in the manufacture of meat products, it is desirable to investigate the effect of faba bean protein on the meat protein matrix under different pH levels. Fundamental data regarding the pH-dependence of functional ingredients and the rheological properties of MP under various pH conditions would be valuable for developing high-quality meat products. However, few studies have been conducted on the application of faba bean to MP under different pH conditions. Therefore, the aim of this study was to evaluate the rheological properties of FBPI-containing pork MP gels (MPGs) under various pH conditions.

## MATERIALS AND METHODS

### Materials

Raw pork loin was purchased from a retail meat market (Gwangju, Korea) to extract the salt-soluble proteins. It was trimmed to remove the connective tissues and external fat, and cut into cubes (1–2 cm^3^). The trimmed pork loin cubes were vacuum-packed and stored frozen at −20°C until required for the experiments. FBPI (90% protein content) was provided by Yantai Shuangta Food.

### Preparation of pork myofibrillar protein gels

After the frozen pork loin cubes were thawed for about 12 h in a refrigerator at 4°C, they were homogenized with 0.1 M sodium chloride (NaCl) and 50 mM sodium phosphate buffer solution (pH 6.25) for 90 s and were centrifuged at 4,800×g for 15 min. After the precipitate was collected, it was homogenized with the buffer solution, and centrifuged; this process was performed three times [11]. Finally, the precipitate was collected, blended with 0.1 M NaCl buffer solution to remove connective tissue, and centrifuged. The protein concentration of MP was determined by the biuret method using a standard curve of bovine serum albumin. Then, the extracted MP was adjusted to a protein concentration of 4% and a salt concentration of 0.45 M by dilution with NaCl and phosphate buffer solution. Control groups and FBPI-treated groups were prepared by the addition of buffer solution adjusted to pH 6.00, 6.25, and 6.50 at 0.45 M salt (Table 1).

### Cooking yield

The cooking yield (CY, %) of pork MPG was determined by heating from 20°C to 80°C in a water bath (WB-22; Daihan Scientific). After heating, the samples were cooled for 12 h in a refrigerator at 4°C and then stored at ambient temperature for 30 min. The CY (%) was calculated by the following formula:


(1) CY (%)=Sample weight after heating (g)/Sample weight before heating (g)×100

### Gel strength of cooked samples

The gel strength (GS, gf) of cooked pork MPG samples was measured using an Instron Universal Testing Machine (Model 3344; Instron) and Merlin software (Instron), according to the method of Lee and Chin [12], to determine the effect of the addition of FBPI (0.5% or 1.0%). The first peak values of the breaking force (gf) were measured by the compression test using a 500 N load cell at a cross speed of 50 mm/min (9 mm diameter).

### Protein surface hydrophobicity of raw samples

The protein surface hydrophobicity (*H*_0_) was measured using the method of Chelh et al. [13]. The MP solution was adjusted to 10 mg/mL of protein concentration with 20 mM sodium phosphate buffer (pH 6.0). Then, 1 mL of MP solution and 200 μL of bromophenol blue solution were mixed; the control was prepared by replacing the MP solution with 1 mL of 20 mM sodium phosphate buffer. The MPG samples and control were agitated with a vortex mixer (KMC-1300V; Vision Scientific) for 10 min at room temperature. Then, they were centrifuged at 2,000×g for 15 min using a VS-5500 centrifuge (Vision Scientific). The optical density (OD) of the samples and control were measured at a wavelength of 595 nm using a spectrophotometer (X-ma 1200; Human). Hydrophobicity values were calculated using the formula:


(2) $$H_{0}\;(\mu \text{g})=200\;\mu \text{L}\times \text{OD of the blank}-\text{OD of the sample}/\text{OD of the blank}$$

### Sulfhydryl group of raw samples

The sulfhydryl content was measured according to a modified version of Ellman’s method [14]. To determine the sulfhydryl groups (-SH, μmol/g), the MP solutions (0.1 g) were mixed with double-distilled water (8.4 mL), 4 mg/mL of 5,5′-dithiobis-2-nitrobenzoic acid solution (0.5 mL), and Tris-Gly-8 M urea (1 mL). The control was prepared by replacing the sample solution with 20 mM phosphate buffer (pH 6.25). After the mixtures were incubated for 5 min at room temperature, the absorbance values were measured using a spectrophotometer at a wavelength of 412 nm. The -SH contents were calculated using a molar extinction coefficient of 13,600 M^−1^ cm^−1^.

### Viscosity of raw samples

The viscosity was determined by measuring the shear stress (Pa) values with increasing shear rate (1/s) in the range of 0 to 600/s using a cylinder-type rotational rheometer (RC30; Rheotec Messtechnik), as described by Lee and Chin [15]. For analysis of the viscosity results, a graph of the viscosity data was constructed using Excel (ver. 2016; Microsoft).

### Sodium dodecyl sulfate-polyacrylamide gel electrophoresis

Sodium dodecyl sulfate-polyacrylamide gel electrophoresis (SDS-PAGE) was performed to determine the protein profiles of FBPI-MPGs under different pH levels, following the method of Laemmli [16]. The samples were adjusted to a 1% protein concentration and loaded on a 10% acrylamide separating gel (0.375 M Tris, pH 8.8) and 4% stacking gel (0.125 M Tris, pH 6.8) with Laemmli sample buffer (Cat. Number 161-0737; Bio-Rad Laboratories). The protein bands were separated by electrophoresis at 150 V for 1.5 h. A standard marker (Cat. Number 161-0318; Bio-Rad Laboratories) was used to investigate the molecular weight of the proteins in the samples.

### Microstructure of cooked samples

The three-dimensional (3D) microstructure of pork MPGs was analyzed according to the method described by Lee and Chin [12]. The microstructural observation of pork MPGs was detected with a low-vacuum scanning electron microscope (JSM-6610LV; JEOL). The samples were cut into cubes of approximately 3 mm^3^ and soaked in 2.5% glutaraldehyde in 0.1 M sodium phosphate buffer (pH 7.0) for 24 h at 4°C for fixing. A 0.1 M sodium phosphate buffer with 1% osmium tetraoxide (OsO_4_) was used to drench the post-fixed samples for 5 h. Then, the samples were dehydrated by washing with 50%, 60%, 70%, 80%, 90%, and 100% ethanol solution and 100% acetone solution for 10 min each. Dehydrated samples were gold-coated using an auto sputter coater (Cressington Scientific Instruments). The microstructure of samples was observed at magnifications of ×1,000 and ×2,000.

### Statistical analysis

All measurements were conducted in triplicate and were reported as mean±standard deviation. A two-way ANOVA (three pH levels×two FBPI levels) was performed using IBM SPSS Statistics 27 (IBM). When no interaction was detected, data were pooled by FBPI addition or by pH level. Duncan’s multiple range test was applied at the 0.05 significance level (95 % confidence) for post-hoc comparisons. Prior to visualization, the dataset was log-transformed, and clustering was performed using Euclidean distance with complete linkage. Correlation patterns across the six treatment groups (C6.00, C6.25, C6.50, F6.00, F6.25, and F6.50) were displayed to compare trends among variables across the different pH conditions.

## RESULTS AND DISCUSSION

### Cooking yield

Table 2 shows the CY (%) of pork MPGs, including FBPI-added treatments at different pH levels. There was no interaction between FBPI addition and pH level on CY (p>0.05). The CYs of pork MPGs containing FBPI were higher than those of the control (p<0.05). These results showed that the addition of FBPI could increase the CY of MPGs. Ananta and Chin [17] reported that the CY of MPGs increased with increased addition levels of cranberry bean protein isolate, leading to the exposure of hydrophilic groups resulting from protein unfolding and the structural entrapment of water within the composite gel network. Glutamic and aspartic acids are hydrophilic and abundantly contained in legumes, such as red bean and faba bean [18,19]. Therefore, protein from legumes might influence the techno-functional properties of foods, and it is considered that the CY increased with the improvement of the WHC.

There were differences in the CY of MPGs under different pH conditions (p<0.05). The CYs of MPGs at pH 6.50 were higher than those at lower pH conditions, and MPGs at pH 6.00 had the lowest CY among all pH conditions (p<0.05). It indicated that the CYs of MPGs increased with increasing pH level, and there was a high correlation between CY and pH level. Hong et al. [5] found no difference between the CYs of porcine MP emulsion gels at pH 6.00 and 6.50, but a higher amount of moisture loss occurred at relatively lower pH conditions (pH 5.00–5.50), regardless of the addition of MTG or calcium alginate. Animal muscle usually has a pI of about pH 5.50; at this pH, electrostatic repulsions between amino acids with opposite charges are minimized [20]. Thus, the water solubility is lowered, and the interaction between water and protein is weakened at the lower pH, so the WHC might be lowered. In the present study, the CY of FBPI-MPGs increased compared to the control groups, and moisture loss decreased with increasing pH levels.

### Gel strength

The GS (gf) of MPGs, including FBPI-added treatments, at different pH values are shown in Table 2. There was no interaction effect of the two factors on GS (p>0.05). GS was decreased in the control groups (without FBPI) compared to the FBPI-added treatments (p<0.05). The GS and WHC are indicative of protein functionalities within meat protein system. Zhuang et al. [21] reported that water exudation from MP gelation can form water cavities on the gel surface, potentially weakening the protein network during heating process. Thus, a higher WHC or CY is associated with increased GS of MP, and these findings could be consistent with the present study.

Several studies have reported that the incorporation of legume proteins could improve the techno-functional properties of MP, including GS. Niu et al. [2] reported that the addition of mild-heated SPI (0.25%, 0.50%, and 0.75%) increased the gel penetration force of chicken MP compared to the control. Kim and Chin [22] reported that the GS of pork MPGs was increased by the addition of chickpea powder compared to the control without formulation. They suggested that the improvement in GS might be associated with the higher total protein content and stronger protein-protein interactions provided by legume proteins, resulting in a protein structure with improved GS. The present study showed similar results to previous studies that researched solubilized meat proteins incorporated with plant-derived proteins. Since legume proteins contain storage proteins, such as 7S and 11S globulins, which can improve gelling properties [23], the addition of FBPI might be suitable for improving the techno-functional properties of processed meats.

GS did not differ among different pH conditions (p>0.05). This suggests that the different pH conditions did not affect the GS of MP protein in this study. However, previous studies reported different results. For example, Zhao et al. [24] reported that the penetration forces of pork MP emulsions at pH 6.50 were higher than those at pH 6.00. Chen et al. [25] demonstrated that the GS of sodium pyrophosphate (SPP)-modified chicken MP-stabilized emulsion gels increased as the pH increased from 5.00 to 6.00, but decreased with further increasing the pH from 6.00 to 9.00. They concluded that the SPP-modified MP emulsion gels had an increased GS and more elastic structure at around pH 6.0 due to an appropriate level of gel formation as a result of protein-protein cross-linking involving ionic interactions between phosphate groups and amino acids. However, Langton et al. [9] reported that no difference in the compression stress of faba bean protein gels were observed between pH 5.00 and 7.00. Although their study did not apply for MP system, their findings indicated that the gel structure derived from faba bean protein composition is not highly responsive to changes in pH. Generally, the maximum GS of MP occurs at around pH 6.00, but differs depending on the muscle fiber type, animal species, and protein concentration [3]. Based on these reports, the techno-functional properties of MPGs, including GS, were not influenced by the pH levels alone, but they may vary depending on the experimental condition.

### Protein surface hydrophobicity

As shown in Table 2, the data were pooled by treatment or pH level as no interaction between FBPI and pH level for the *H*_0_ of MPGs was observed (p>0.05). The *H*_0_ values of FBPI-added treatments were higher than those of the controls (p<0.05). Protein structure has the properties of forming aggregates by hydrophobic interactions [26]. Increased hydrophobicity leads to exposure of non-polar amino acids in the protein structure, which can be associated with changes in protein interactions relevant to gelation and emulsification [27]. These reports indicated that increased hydrophobicity due to the addition of FBPI into MPGs might induce structural changes in the protein matrix, resulting in improved techno-functional properties. Chen et al. [28] reported that a mixture of large yellow croaker MP and pea protein showed higher hydrophobicity than MP alone. Additionally, *H*_0_ was increased when pea protein, pretreated to expose a large proportion of the hydrophobic groups in the protein structure, was incorporated into soluble muscle protein extract [29]. Thus, proteins from legumes, such as faba bean and pea, improved the hydrophobicity due to the presence of globulins, including conglycinin and glycinin.

The *H*_0_ values of MPGs at pH 6.25 were not different from those at either pH 6.00 or 6.50 (p>0.05), whereas there was an upward trend in the *H*_0_ of MPGs from pH 6.00 to 6.50. Jiang et al. [30] reported that the *H*_0_ values of SPI were higher at pH 11.0–12.0 than at pH 1.5–2.5. They suggested that increased pH improved the techno-functional properties, such as surface activity and emulsifying capacity, due to exposure of glycinin and induction of protein aggregation. In general, there is a strong correlation between the solubility and *H*_0_ of MP [31]. Protein solubility reaches a minimum at the pI, and the solubility can be improved when the pH is adjusted to an appropriate level [32]. Thus, the *H*_0_ of MPGs was affected by the pH level due to the changes in protein solubility and structure in this study.

### Sulfhydryl groups

The -SH contents of MPGs, including FBPI-added treatments at various pH levels, are shown in Table 2. There was no interaction effect between FBPI and pH on *H*_0_ (p*>*0.05). The FBPI-treated groups had lower -SH contents than the controls (p<0.05), suggesting that the incorporation of FBPI induced a decrease in the -SH content of MPGs. The -SH affected the protein structure and redox mechanism, and performed an important function in maintaining protein formation [33]. Decreased -SH indicated increased disulfide (S-S) bonds enhanced by exposing those inside myosin of MP [34]. Liu et al. [35] reported that the addition of SPI decreased the -SH content in chicken MP due to the 7S and 11S globulins in legume proteins. Plant-based protein can increase hydrophobic interaction and disulfide bonds. This is because the helical structure in the myosin molecule unfolds during the heating of the protein, exposing hydrophobic amino acids and SH groups by hydrophobic forces and disulfide bonds [36].

No differences in the content of -SH were observed among different pH levels (p>0.05). Lu et al. [4] reported that pH-shifting from alkaline to neutral could modify the secondary structure of chicken MP by exposing reactive -SH. Marmon et al. [20] reported that MP showed increased hydrophobic interactions with increasing sulfhydryl bridges and ionic bonds at pH 5.5 more than at pH 6.5. Based on the results, the -SH content of MPs may have various trends depending on the range of pH or the animal species from which the MPs were derived, and a noticeable change was not observed in this study.

### Viscosity

Figure 1 shows the shear stress values with increasing shear rate of MPGs including FBPI-added treatments at different pH levels. The shear stress values of FBPI-treated group had similar trends at all pH levels and tended to be higher than the controls at all shear rates. In contrast, there were big changes among the controls across different pH conditions. At pH 6.50, the control showed higher shear stress values compared to other pH levels, while those at pH 6.00 were the lowest among all pH conditions studied. These viscosity results demonstrated that shear stress in the control group is strongly dependent on pH, whereas FBPI addition stabilized shear stress across pH conditions. Cofrades et al. [37] reported that an increase in pH increased the apparent viscosity of actomyosin from chicken, pork, and beef. It is known that the pH-dependent increase in viscosity was attributed to molecular swelling within muscle fiber fragments [38]. The incorporation of legume proteins can bind water in the protein structure, resulting in the improvement of viscoelastic abilities [39]. Furthermore, globulin is able to enhance the viscosity of protein structure through crystallization (mesophase) in formation [40]. Because faba bean contains globulins, which increase the viscosity of the protein matrix, FBPI-treated groups showed higher shear stress values than those of control groups. Therefore, FBPI addition into a pork MPG could increase the viscosity over a certain range of shear stress, without being affected by the pH levels, compared to the control groups without FBPI.

### Sodium dodecyl sulfate-polyacrylamide electrophoresis

Figure 2 shows the SDS-PAGE results of MPGs, including FBPI-added treatments at different pH levels. No differences in the protein profiles were observed among the control and treated groups at different pH levels. In the protein profiles of FBPI-added treatments, two bands that did not appear in the controls were detected at ~42 and 50 kDa, and the intensities of those bands slightly increased with increasing pH level. Bühler et al. [41] reported that convicilin, vicilin, and α-legumin presented at 60, 46–55, and 38–40 kDa in the protein patterns of faba bean protein concentrate, respectively. Sun and Arntfield [42] reported that bands corresponding to the vicilin and legumin subunits were observed in the electrophoretic pattern of a mixture of chicken MP with pea protein. It is known that the legumin proteins can form a protein network supported by hydrogen bonds and hydrophobic interactions [43]. Wagner and Sorgentini [26] reported that the protein fraction corresponding to the 11S subunit gradually decreased as it migrated to insoluble aggregates at lower pH in the protein patterns of SPI. The solubility of legumin and vicilin (11S and 7S globulins) derived from faba bean protein was closely related to the pH condition, and 11S globulin was very insoluble with a lowering of pH, regardless of legume species [9,10]. In this study, globulin fractions, which can structurally enhance the protein matrix, were detected due to the presence of FBPI in the protein profiles of MPGs, and different pH levels might affect the band intensities of globulins due to the correlation between pH and solubility.

### Microstructure

Figure 3 illustrates the microstructures of MPGs, including FBPI-added treatments at the different pH values. At all pH conditions (pH 6.00–6.50), the microstructures of FBPI-treated groups had a less porous and flatter surface, resulting in a denser and smoother appearance compared to the control groups, which showed heterogeneous particles and many pores under the same pH conditions. In these micrographs, FBPI might be combined well in MPGs, filling some of the pores that were more noticeable in the control groups. Borderías et al. [44] concluded that the homogenous structure of pollack MP and the porosity seem to be reduced as the content of pea protein isolate was increased. These results suggested that the presence of proteins from legumes, such as pea, black bean, mung bean, and faba bean, in the MP matrix developed the desirable structure by reinforcing protein-protein interactions and cross-linking.

In the control groups, the micrographs of MPGs at pH 6.00 were similar to those at pH 6.25. Under the pH 6.50 condition, the surface of MPG appeared more compact and denser than at lower pH levels. When FBPI is incorporated into MPGs, the differences depending on pH levels seem to be more pronounced. The surface of FBPI-added treatments had decreased porosity, and its compactness and homogeneity appeared to increase as the pH increased, resulting in a well-structured and interconnected network at pH 6.50. Hong et al. [5] reported that the optical microstructures of pork MP at pH 6.50 had smaller emulsion droplets than those at pH 5.00, regardless of the addition of MTGase or calcium alginate. Zhao et al. [24] concluded that micrographs of MPG at pH 6.50 showed a more compact and uniform 3D protein network than those at pH 6.00. They explained that the net charge effect at higher pH level induced an unfolding of the structure of MP and exposed hydrophilic amino acids, resulting in orderly aggregation [45].

### Correlation among functional and structural properties

As shown in Figure 4, the correlation analysis demonstrated relationships among CY, GS, *H*_0_, -SH groups in MPGs formulated with and without FBPI under different pH conditions. The CY exhibited a strong positive correlation with the GS, indicating that improved water retention was closely associated with the development of firmer gel network. This trend was more pronounced in FBPI-treated groups, suggesting that the incorporation of FBPI supported gel formation similar to the enhancement observed at higher pH levels. The *H*_0_ showed a positive association with GS, indicating that the exposure of hydrophobic residues can promote protein–protein interactions that contributed to gel formation. In contrast, -SH groups presented negative correlations with both GS and *H*_0_. These relationships indicated that a reduction in -SH groups was accompanied by increased disulfide bond formations, which stabilized the gel network. Consequently, the observed correlations suggested that FBPI addition promoted structural changes that were consistent across the different pH conditions, contributing to the improvement of gel structure.

## CONCLUSION

FBPI addition to pork MPGs increased the CY (90.6 vs. 87.7%), *H*_0_ (33.5 vs. 24.9 μg), while decreasing the -SH content (12.0 vs. 16.0 μmol/g) compared to the control, regardless of the pH conditions. Since no interaction between pH and FBPI on the CY, GS, *H*_0_, and -SH content of MPG was observed, various pH conditions did not affect the ability of FBPI to improve the techno-functional properties of salt-soluble proteins. The shear stress curves of FBPI-treated groups tended to be higher than those of the control groups over most shear rates. In addition, FBPI treatments showed overlapping shear stress curves across the different pH levels, whereas the control groups showed visible separation between curves as the pH changed. Furthermore, in the SDS-PAGE protein patterns and microstructure images, the band intensity of the globulins increased and the microstructure appeared flatter and more compact as the pH increased (6.00–6.50). Therefore, FBPI could be applied to meat protein systems as a functional ingredient that provided generally consistent effects across the various pH values.

## Figures and Tables

**Figure 1:**
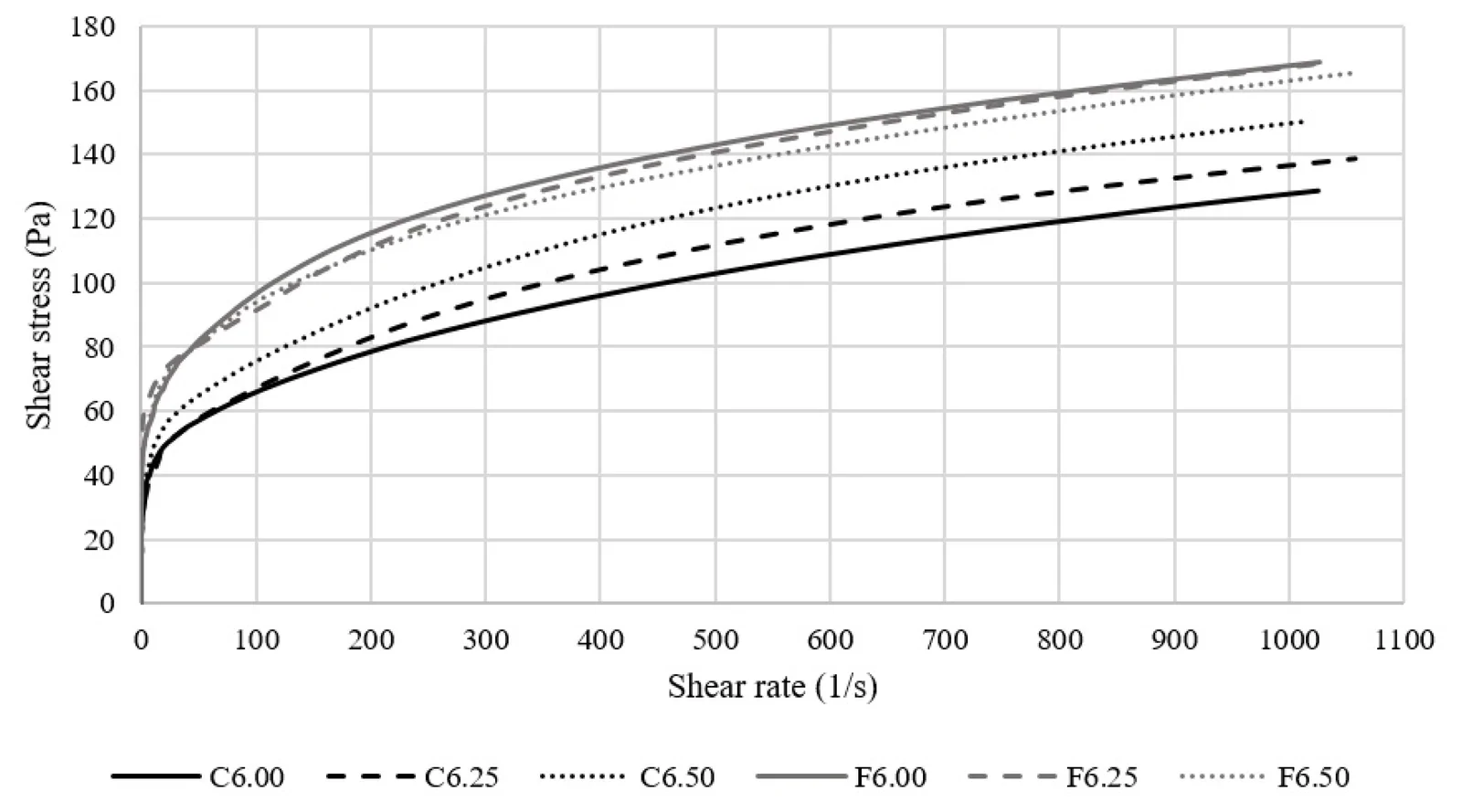
Viscosity of pork myofibrillar protein gels (MPGs) with faba bean protein isolate (FBPI) as affected by different pH levels. Treatments: C6.00, MPG adjusted to pH 6.00; C6.25, MPG adjusted to pH 6.25; C6.50, MPG adjusted to pH 6.50; F6.00, MPG adjusted to pH 6.00 with 1.0% FBPI; F6.25, MPG adjusted to pH 6.25 with 1.0% FBPI; F6.50, MPG adjusted to pH 6.50 with 1.0% FBPI.

**Figure 2:**
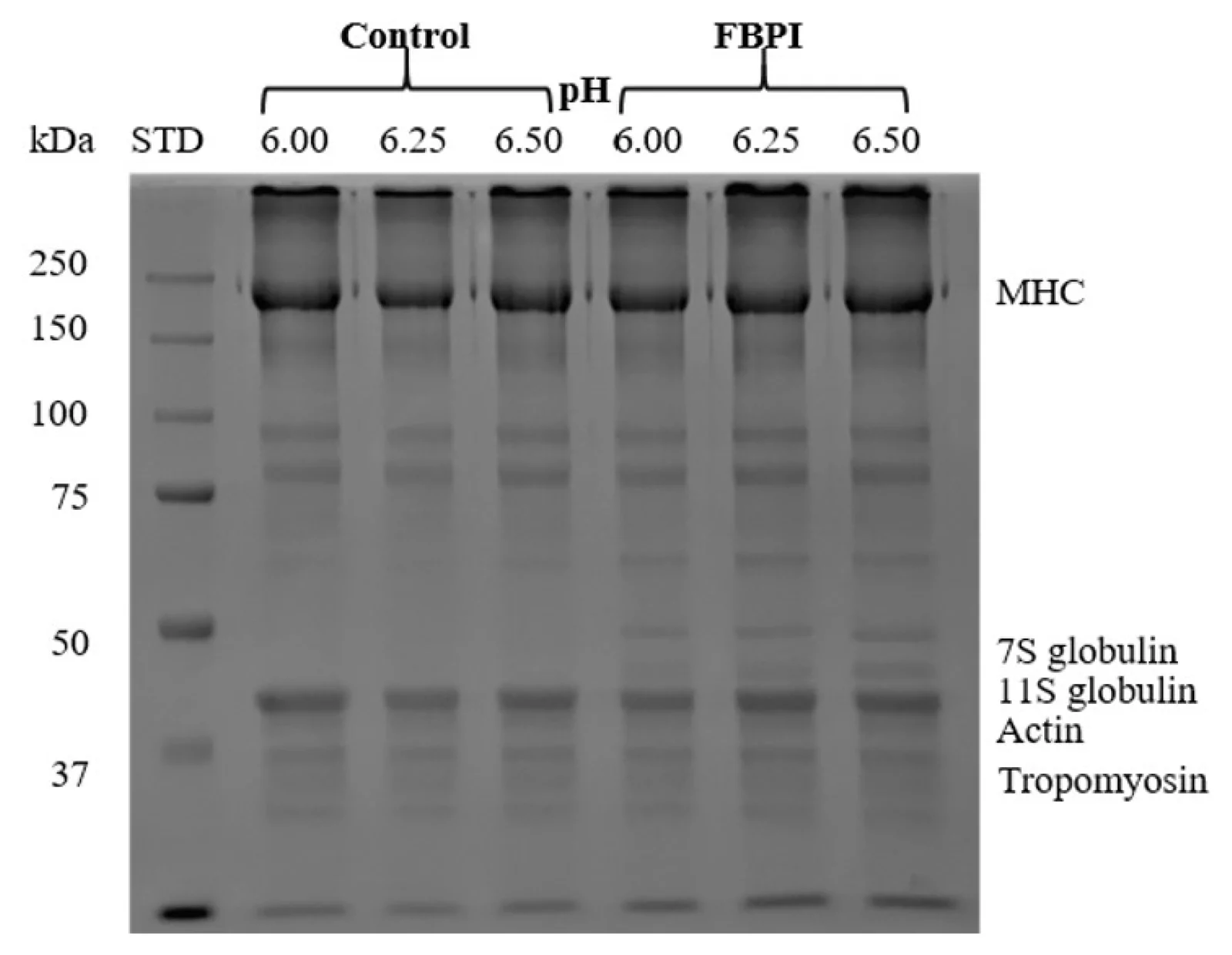
Sodium dodecyl sulfate-polyacrylamide gel electrophoresis profile of myofibrillar protein gels (MPGs) with faba bean protein isolate (FBPI) as affected by different pH levels. STD: protein standard marker. Treatments: C6.00, MPG adjusted to pH 6.00; C6.25, MPG adjusted to pH 6.25; C6.50, MPG adjusted to pH 6.50; F6.00, MPG adjusted to pH 6.00 with 1.0% FBPI; F6.25, MPG adjusted to pH 6.25 with 1.0% FBPI; F6.50, MPG adjusted to pH 6.50 with 1.0% FBPI.

**Figure 3:**
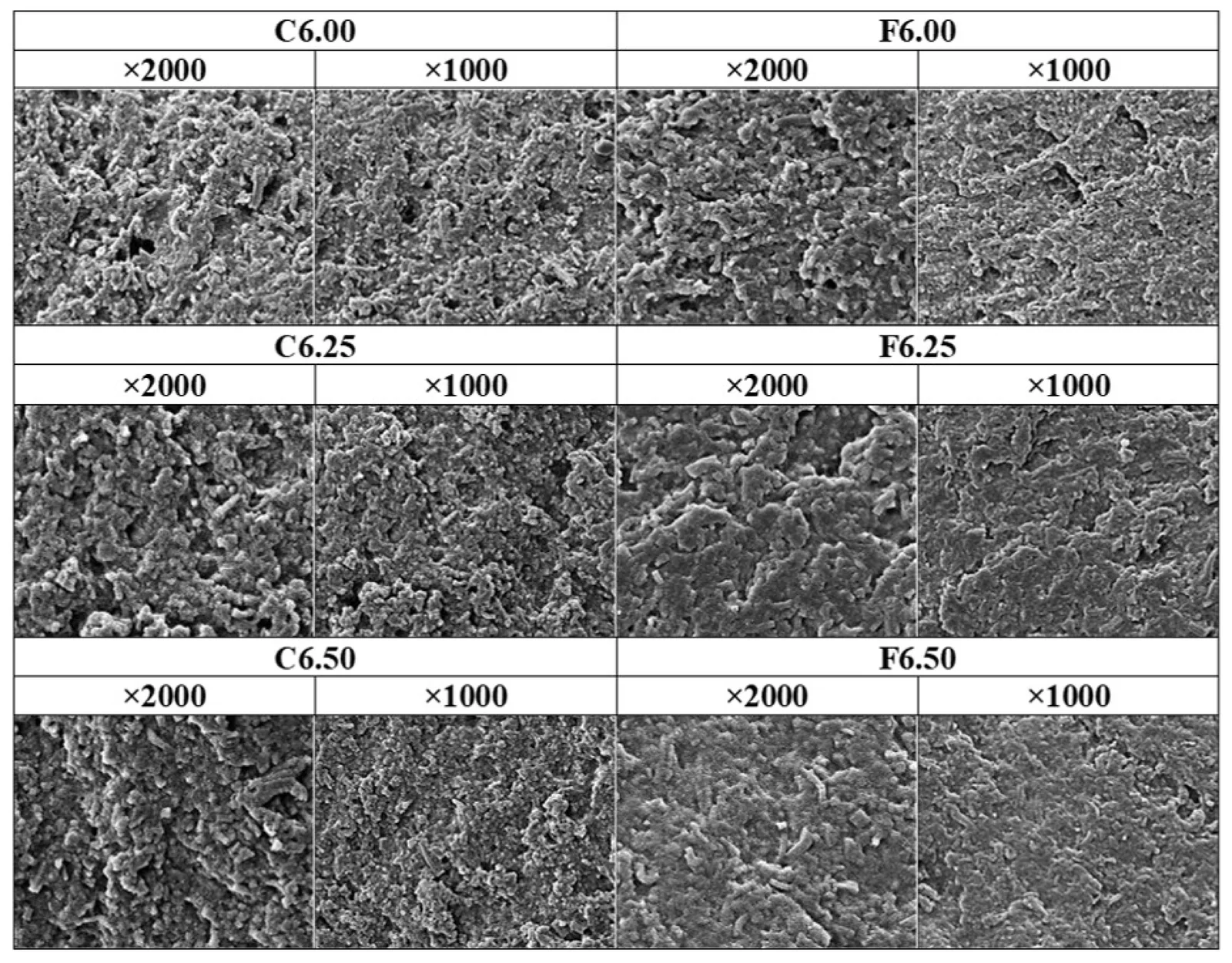
Microstructure of myofibrillar protein gels (MPGs) with faba bean protein isolate (FBPI) as affected by different pH levels. Treatments: C6.00, MPG adjusted to pH 6.00; C6.25, MPG adjusted to pH 6.25; C6.50, MPG adjusted to pH 6.50; F6.00, MPG adjusted to pH 6.00 with 1.0% FBPI; F6.25, MPG adjusted to pH 6.25 with 1.0% FBPI; F6.50, MPG adjusted to pH 6.50 with 1.0% FBPI.

**Figure 4:**
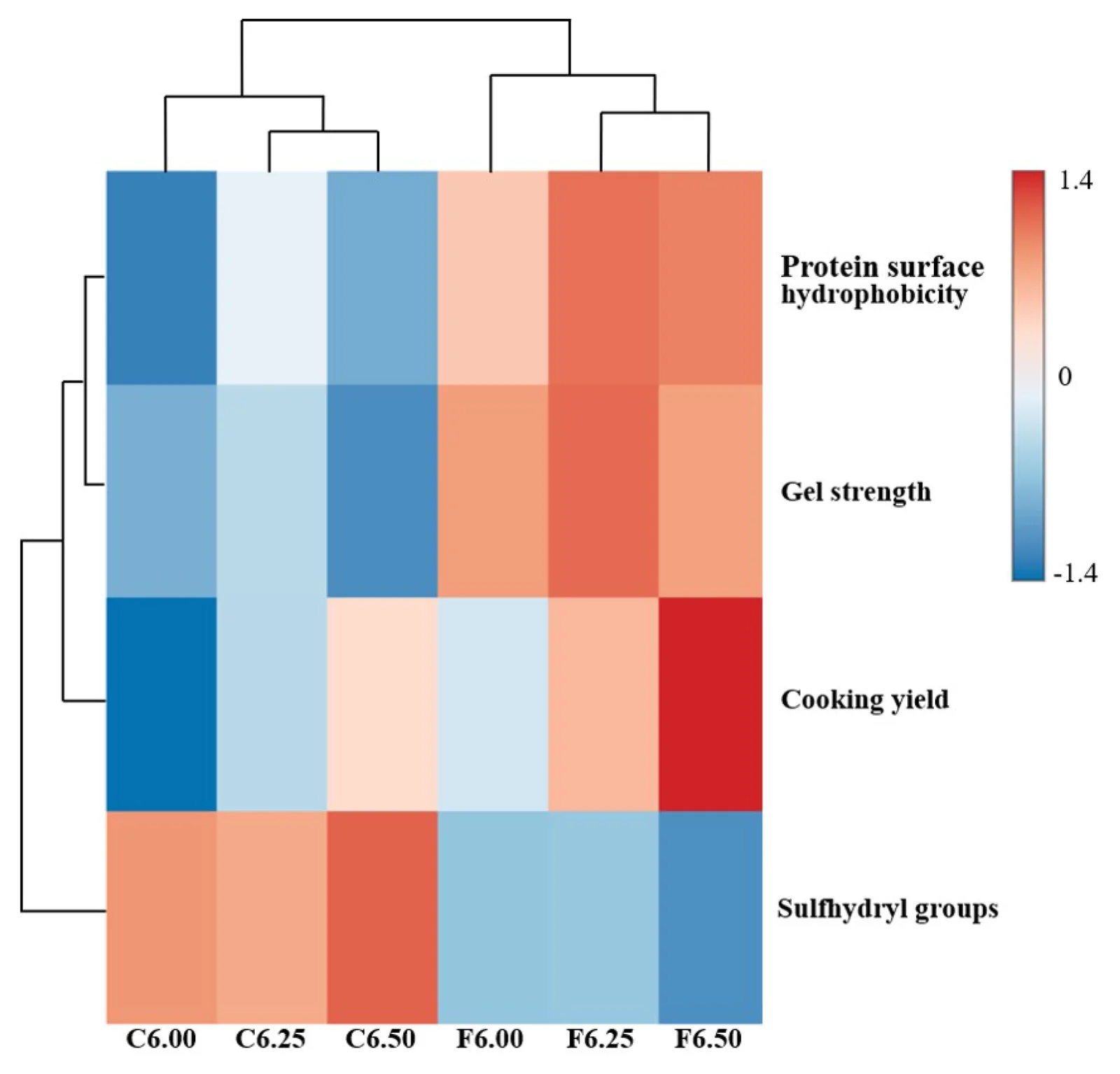
Correlation heatmap of functional parameters in pork myofibrillar protein gels (MPGs) as affected by the addition of faba bean protein isolate (FBPI) and pH conditions. Treatments: C6.00, MPG adjusted to pH 6.00; C6.25, MPG adjusted to pH 6.25; C6.50, MPG adjusted to pH 6.50; F6.00, MPG formulated to pH 6.00 with 1.0% FBPI; F6.25, MPG formulated to pH 6.25 with 1.0% FBPI; F6.50, MPG formulated to pH 6.50 with 1.0% FBPI. Red reflects positive correlation coefficients, whereas blue reflects negative coefficients.

**Table 1:** Formulation of pork myofibrillar protein (MP) gels with faba bean protein isolate (FBPI) at different pH levels

Ingredients (%)	Control			FBPI		

	pH levels					

	6.00	6.25	6.50	6.00	6.25	6.50
MP+0.45 M NaCl buffer	80.0	80.0	80.0	80.0	80.0	80.0
50 mM NaH_2_PO_4_ buffer	20.0	20.0	20.0	19.0	19.0	19.0
FBPI	-	-	-	1.00	1.00	1.00
Total	100.0	100.0	100.0	100.0	100.0	100.0

**Table 2:** Cooking yield (CY), gel strength (GS), protein surface hydrophobicity (H_0_), and sulfhydryl (-SH) content of myofibrillar protein gels with faba bean protein isolate (FBPI) at different pH levels

	CY (%)	GS (gf)	H_0_ (μg)	-SH (μmol/g)
Treatments				
Control	87.7±2.43^b^	99.5±7.92^b^	24.9±3.67^b^	16.0±0.67^a^
FBPI	90.6±0.72^a^	135±17.2^a^	33.5±2.32^a^	12.0±1.60^b^
pH				
6.00	86.9±2.26^c^	115±20.4^a^	26.9±5.23^b^	14.1±2.25^a^
6.25	89.2±1.67^b^	123±21.9^a^	29.2±6.41^ab^	13.9±1.87^a^
6.50	91.3±1.84^a^	118±26.9^a^	31.3±4.09^a^	14.0±3.27^a^

a–c Means having the same superscripts in the same column are not different (p>0.05).

## Data Availability

Upon reasonable request, the datasets of this study can be available from the corresponding author.
